# A New Pattern Quality Assessment Criterion and Defocusing Degree Determination of Laser Speckle Correlation Method

**DOI:** 10.3390/s21144728

**Published:** 2021-07-10

**Authors:** Wenxin Hu, Zhipeng Sheng, Keyu Yan, Hong Miao, Yu Fu

**Affiliations:** 1 College of Physics and Optoelectronic Engineering, Shenzhen University, 3688 Nanhai Avenue, Shenzhen 518060, China; huwenxin@szu.edu.cn (W.H.); shengzhipeng1993@163.com (Z.S.); yankeyu@szu.edu.cn (K.Y.); 2 CAS Key Laboratory of Mechanical Behavior and Design of Materials, Department of Modern Mechanics, University of Science and Technology of China, Hefei 230027, China; miaohong@ustc.edu.cn

**Keywords:** laser speckle correlation, defocusing degree, speckle pattern quality criterion, vibration measurement, rotation measurement

## Abstract

The laser speckle correlation method has found widespread application for obtaining information from vibrating objects. However, the resolution and accuracy of the laser speckle correlation method as they relate to the defocusing degree have not been analyzed sufficiently. Furthermore, the possible methods for speckle pattern quality assessment and enhancement have not been studied. In this study, the resolution and accuracy of the laser speckle correlation method are analyzed, and it is found that they are affected by the defocusing degree and speckle pattern quality, respectively. A new speckle pattern quality criterion combining the mean intensity gradient and frequency spectrum was proposed, called CMZ. The quality of the speckle pattern is higher when the CMZ is closer to zero. The proposed criterion was verified by simulated speckle patterns and real speckle patterns with different speckle sizes, densities, and gray contrasts. In the experimental setup stage, a suitable defocusing degree can be selected based on the resolution requirement and optimal speckle size, and other experimental parameters can be determined according to the CMZ criterion. Rotation and vibration experiments verified the effectiveness of the laser speckle correlation method and confirmed the reliability of the experiment preparation based on proposed CMZ criterion.

## 1. Introduction

Optical dynamic measurements have been widely used to detect noncontact vibrations, continuous deformation, or movement of objects in various research and industrial applications. The common methods are divided into interferometric and imaging-based methods. Interferometric methods include electric speckle pattern interferometry (ESPI) [1,2,3,4], shear interferometry [5,6], and holographic interferometry [7,8,9], and these methods generally produce subwavelength accuracy. Adopting high-efficiency phase extraction methods, such as the temporal phase-shifting method [10,11], spatial carrier phase-shifting method [12,13], and Fourier transform method [14,15], nanometer accuracy can be achieved under laboratory conditions. However, its applications are limited by the sampling rate of camera and environmental requirements. The more powerful laser Doppler vibrometry (LDV) technique [16,17,18] can provide single-point high-speed dynamic measurements using a photoelectric detector, but it is still essentially an interferometer that is sensitive to environmental fluctuations. Furthermore, the digital image correlation (DIC) method [19,20,21] is sensitive to object surface displacement, especially in-plane displacement. In DIC, artificial speckle or laser speckle is as a carrier of deformation information and deforms together with the specimen surface. Artificial speckle is most commonly used and is usually prepared by spraying paints on the sample surface [22] or transferring speckle patterns to sample surface using the water transfer printing technique [23]. However, laser speckles, formed by the reflection and scattering of laser irradiation onto a rough surface, are desirable in some situations. For example, in a high temperature environment, artificial speckle will inevitably fall off and discoloration will occur. Song et al. [24] and Zheng et al. [25] have applied laser speckle correlation method to a high temperature measurement field successfully.

Gregory et al. [26] proposed defocused speckle photography and described how to separate tilt (differential) topology variations on a scatter surface from linear displacements. Horváth et al. [27] deduced the relationship between the small-deformation tensor and the speckle field displacement in detail. Based on these analyses, another simple and prospective application of the laser speckle correlation method has emerged. Jo et al. [28] proposes to observe the movement of the secondary speckle patterns that are generated on top of a target when it is illuminated by a laser beam spot. Through proper defocusing, the movement of the object creates a scenario in which the same speckle pattern moves or vibrates in the transverse plane, instead of the speckle pattern constantly changing. Gradually, this method has achieved widespread application for obtaining information from vibrating objects. For instance, Zeev et al. [29], Lin et al. [30], and Yevgeny et al. [31] applied this method for the simultaneous remote extraction of multiple speech sources, vibration measurements, and blood pulse pressure measurements, respectively. Furthermore, Wu et al. [32] introduced a high-speed optical flow algorithm to tracking laser speckle images to realize real-time audio detection and regeneration of a moving sound source. However, the influence of the laser speckle quality and possible ways to achieve quality assessment and enhancement have not received enough attention in vibration measurements. Furthermore, the key parameter defocusing degree can be selected combining optimal speckle pattern quality and resolution requirement.

In the DIC field, many quality assessment criteria aimed at sprayed speckle patterns have been developed gradually. Subset entropy [33] and the sum of square subset intensity gradients (SSSIG) [34] are suitable for subset optimization. In order to evaluate the quality of the whole speckle pattern, Lecompte et al. [35] first proposed the mean speckle size based on the image morphology, and then Grammond et al. [36] applied edge detection to determine the speckle size and density. These methods based on speckle morphology lack the ability to evaluate gray information, such as the contrast influence on the speckle pattern quality. To overcome this deficiency, the mean intensity gradient (MIG) [37], the mean intensity of the second derivative (MIOSD) [38] and standard deviation of gray intensities within each speckle (SDGIS) [39] are proposed successively. Another trend is to consider the primary and secondary peaks of the autocorrelation functions [40,41,42]. However, compared with the sprayed speckle, the laser speckle has a more uniform distribution of speckle particles, a smaller difference of the gray standard deviations between individual speckles, and non-obvious secondary auto-correlation peaks. Thus, the assessment criteria described above cannot be used directly. Song et al. proposed a new index, the multi-factor fusion index (MFFI) [43], which took the inhomogeneity of the gray contribution, the mean square deviation of the gray contribution, and the standard deviation of the speckle particles size into consideration.

In this study, the resolution of the laser speckle correlation method is analyzed, and its main influence factors are distance relationships between the measurement planes, which depend on the defocusing degree. The defocusing degree also affects the speckle pattern quality, further influencing the accuracy of the laser speckle correlation method. To ensure a high quality of the speckle pattern, a new speckle pattern quality criterion combining the MIG and frequency spectrum was proposed, called CMZ, which accounts for both random error and the interpolation bias. A simple rule is presented based on the balance of random error and interpolation bias, and it was verified that the quality of the speckle pattern is higher when CMZ is closer to zero. Furthermore, the particular characteristics of the laser speckle have been used, which were distinguished using traditional indices, such as MIG and MIOSD. The proposed criterion was demonstrated by simulated speckle patterns with different speckle sizes and densities. Experimental speckle patterns at different defocusing degrees, exposure times, and measurement locations and the corresponding translation patterns were then used to validate the proposed criterion. Based on the sufficient resolution requirement and optimal speckle size, a suitable defocusing degree (such that the distance relationships between the measurement planes can be determined) and other experimental parameters can be determined according to the CMZ criterion during the experimental setup. Rotation experiments were used to illustrate the relationship between the resolution and the defocusing degree, which further verified the CMZ criterion. Vibration experiments simultaneously verified the effectiveness of the laser speckle correlation method and the reliability of the experimental setup based on the proposed CMZ criterion.

## 2. Defocusing Degree Determination of Laser Speckle Correlation Method

By illuminating an object with a laser beam spot, a speckle pattern can be generated due to the roughness of the object surface. When a spatially coherent beam is reflected from the object whose roughness generates a random phase distribution, we may obtain the self interfering speckle pattern in the far field.

**Figure 1:**
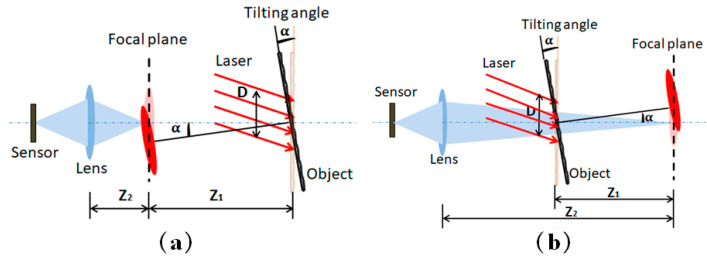
Schematic of the system: camera is focused on the plane (**a**) behind or (**b**) in front of the object.

As shown in Figure 1a,b the camera is focused on the plane behind or in front of the object such that the object itself is defocused, respectively, where the focal plane is at a distance of $Z_{1}$.

According to the analysis conducted by Zeeval et al. [29], this system was sensitive to the tilt, and the effect caused by transversal and axial movement is negligible. When slightly defocusing, object tilt creates a situation in which the same speckle pattern only moves or vibrates in the transverse plane instead of constantly changing the speckle pattern. Thus, shifts of the speckle pattern due to tilt can be easily detected by spatial pattern correlation. According to the geometric relation, the tilt angle α can then be determined as follows:

(1) $$\alpha =\frac{Z_{1}U}{M}$$

where U represents the displacement of the pattern on the camera. M is the imaging system magnification. Once tilt angles along the time axis are obtained during vibration, vibration information including frequency and strain can be calculated. Thus, accurate correlation tracking is a prerequisite to tilt angle calculation, even to vibration analysis.

Valid correlation calculations require a suitable speckle size to be imaged to the sensor plane. In the case of an objective laser speckle, the speckle size S is described as follows:

(2) $$S\approx \frac{{\lambda Z}_{1}}{D}$$

where λ and D are the optical wavelength and the dimension of the illuminated spot, respectively. The size $S^{\prime }$. of the speckle imaged to the sensor plane, which is obtained at the $Z_{1}$ plane, is expressed as follows:

(3) $$S^{\prime }=\frac{{\lambda Z}_{1}}{\mathrm{DM}}$$

To ensure that every speckle in the sensor plane equals K pixels, the condition is described as follows:

(4) $$\frac{{\lambda Z}_{1}}{\mathrm{DM}}=K\times L_{x}$$

where $L_{x}$ is the physical size of one pixel in the CCD sensor.

According to formula (1), a greater distance $Z_{1}$ and a smaller magnification factor M correspond to a higher tilt angle resolution U. When the camera magnification is fixed, the larger $Z_{1}$ corresponds to larger angle resolution U in situations (a) and (b). However, usually the distance between the sensor plane and object is fixed, but the defocusing degree can be adjusted by changing the focal length. When the adjusted parameter is the defocusing degree, the rule is different in the two situations. If the camera is focused on the plane behind the object, as in situation (a), a greater distance $Z_{1}$ means a shorter object distance $Z_{2}$, that is, a smaller magnification factor M and a higher angle resolution U. For situation (b), a greater distance $Z_{1}$ corresponds to a greater magnification factor M, so the variation of $\frac{Z_{1}}{M}$ cannot be judge directly, causing non-determinacy of the angle resolution change. Thus, a reasonable defocusing degree that determines the relative distances between the object, the focal plane, and the sensor plane can be obtained according to speckle size requirement imaged to the sensor plane and a sufficient tilt angle resolution requirement.

## 3. Laser Speckle Pattern Quality Assessment

An effective speckle pattern quality assessment criterion is a prerequisite to ensure correlation tracking. In general, correlation calculation error consists of random error and interpolation bias. Random error highly depends on image noise, which is related to the gray scale of the image. For zero- and first-order shape functions, Pan et al. [34] pointed out that the random error Std is defined as follows:

(5) $$\mathrm{Std}=\frac{\sigma }{\sqrt{\sum_{i=-N}^{N}\sum_{j=-N}^{N}\frac{{\left(g_{x}\left[i,j\right]\right)}^{2}+{\left(g_{y}\left[i,j\right]\right)}^{2}}{2}}}$$

where N is half of the size of the subset, σ is the standard deviation of the image noise. $g_{x}\left[i,j\right]$ and $g_{y}\left[i,j\right]$ are the x- and y-directional gray derivatives at point [i,j], respectively. Su et al. [44] introduced interpolation bias kernel to characterize the frequency response of the interpolation bias, and interpolation bias kernel was defined as

(6) $$E\left(v_{x},v_{y}\right)=\left(v_{x}-1\right)\varphi \left(v_{x}-1,v_{y}\right)-\left(v_{x}+1\right)\varphi \left(v_{x}+1,v_{y}\right)+\varphi \left(v_{x},v_{y}\right)\left(v_{x}+\;1,v_{y}\right)+\varphi \left(v_{x},v_{y}\right)\left(v_{x}-1,v_{y}\right)$$

where $\varphi \left(v_{x},v_{y}\right)$ represents interpolation function (cubic BSpline) at frequency $\left(v_{x},v_{y}\right)$. The result curve of interpolation bias kernel verified that high-frequency components are the major source of interpolation bias.

As for sprayed speckle patterns assessment, MIG and MIOSD are the most commonly used. MIG is defined as

(7) $$\mathrm{MIG}=\frac{\sum_{i=1}^{W}\sum_{j=1}^{H}\sqrt{g_{x}{\left(x\right)}_{\mathrm{ij}}{}^{2}+g_{y}{\left(x\right)}_{\mathrm{ij}}{}^{2}}}{W\times H}$$

MIOSD is defined as:

(8) $$\mathrm{MIOSD}=\frac{\sum_{i=1}^{W}\sum_{j=1}^{H}\sqrt{g_{\mathrm{xx}}{\left(x\right)}_{\mathrm{ij}}{}^{2}+g_{\mathrm{yy}}{\left(x\right)}_{\mathrm{ij}}{}^{2}}}{W\times H}$$

where $g_{x}{\left(x\right)}_{\mathrm{ij}}$ and $g_{y}{\left(x\right)}_{\mathrm{ij}}$ are the x- and y-directional gray derivatives at position $x_{\mathrm{ij}}$, respectively. $g_{\mathrm{xx}}{\left(x\right)}_{\mathrm{ij}}$ and $g_{\mathrm{yy}}{\left(x\right)}_{\mathrm{ij}}$ are the x- and y-directional intensity of the second derivatives at position $x_{\mathrm{ij}}$, respectively. W and H represent the pixel width and pixel height, respectively.

The MIG and MIOSD are defined according to gray gradient, which are supposed to assess random error sufficiently. However, the other component interpolation bias is not only related to the gray gradient. Typically, MIG should be large and continue increasing as the speckle particle size decreases, but this does not mean the smallest speckle particle size is optimal. Based on the rich research in the DIC field, the optimal speckle size is 3–5 pixels [41,42,43,44]. If the speckle size is too small, it leads to image under-sampling, which causes a large interpolation bias. If the speckle size is too large, the details of the image are not rich enough, and the contrast is poor, resulting in a large random error.

Considering high-frequency components are the major source of interpolation bias, we present a new concept called the zero spectrum ratio (ZSR) to quantize frequency spectrum component. The ZSR is defined as

(9) $$\mathrm{ZSR}=\frac{\max\left[\mathrm{FFT}\left(g\right)\right]}{\mathrm{sum}\left[\mathrm{FFT}\left(g\right)\right]}$$

where FFT(f) represents the Fourier transform of the speckle pattern, and max[FFT(g)] and sum[FFT(g)] are the maximum and sum of the frequency spectrum, respectively. The value of ZSR represents the proportion of the zero-order spectrum, which is designed to be related to interpolation bias.

To take both the interpolation error and the random error into account, we propose to combine these two indexes. Because laser speckles have a more uniform distribution of speckle particles and a smaller difference of the gray standard deviation between individual speckle particles, we first analyze the speckle particle size and speckle density effects on the MIG and the ZSR. A speckle density of 100% means that adjacent speckle particles are in contact with each other.

**Figure 2:**
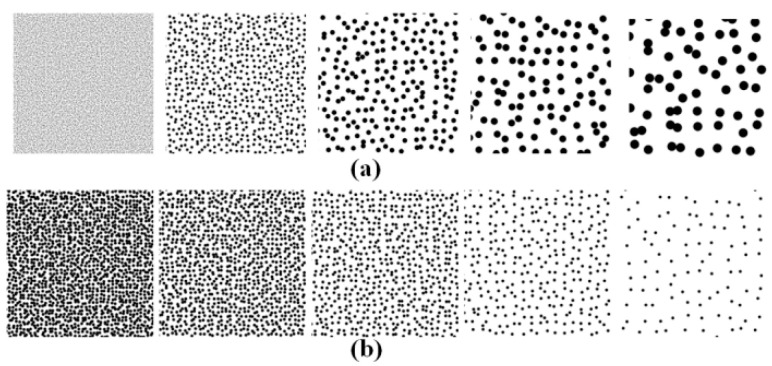
Simulated speckle patterns with (**a**) different speckle sizes and (**b**) different speckle densities.

As shown in Figure 2, we simulated two series of speckle patterns. In series (a), the speckle size increased from 2 to 14 pixels, and the speckle density remained at 50%. In series (b), the speckle size was unchanged, but the speckle density decreased from 80% to 20%. Although real speckle patterns for surfaces could have significantly different appearances [45], this simple model was used to illustrate the proposed quality assessment criterion, then real experiment speckle patterns were applied to ensure its validation.

**Figure 3:**
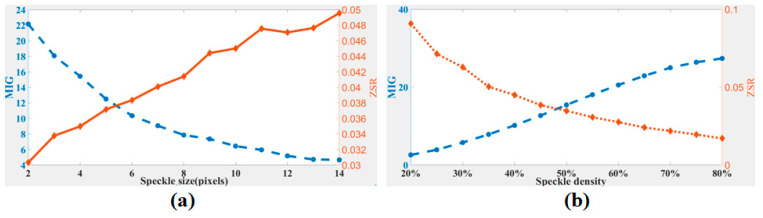
Assessment results of speckle patterns with (**a**) different speckle sizes and (**b**) different speckle densities.

Figure 3a,b give the results of the speckle pattern series (a) and (b), respectively. We found that the values of MIG and ZSR undergo opposite changes with the speckle size increasing or the speckle density decreasing. We propose a new assessment criterion: the speckle pattern quality is higher when the normalized MIG and normalized ZSR are closer to the same value, which can also be understood as finding the intersection of the MIG and ZSR curves. Thus, neither MIG nor ZSR is too big, balancing the interpolation bias and random error. Based on this idea, a new parameter combining the MIG and the ZSR, named CMZ, is defined as

(10) $$\mathrm{CMZ}=\left|{\mathrm{MIG}}^{\prime }-{\mathrm{ZSR}}^{\prime }\right|$$

(11) $${\mathrm{MIG}}^{\prime }=\{\begin{array}{c} 1\;\mathrm{If}\;\mathrm{MIG}>35\\ \frac{\mathrm{MIG}-5}{30}\;\mathrm{If}\;5<\mathrm{MIG}<35\\ 0\;\mathrm{If}\;\mathrm{MIG}<5 \end{array}$$

(12) $${\mathrm{ZSR}}^{\prime }=\{\begin{array}{c} 1\;\mathrm{If}\;\mathrm{ZSR}>0.08\\ \frac{\mathrm{ZSR}-0.01}{0.07}\;\mathrm{If}\;0.01<\mathrm{ZSR}<0.08\\ 0\;\mathrm{If}\;\mathrm{ZSR}<0.01 \end{array}$$

where MIG′ and ZSR′ are the normalized MIG and normalized ZSR, respectively. The ranges of MIG and ZSR need to be determined in advance. Considering the limiting case, the density and the size of speckle were set to be 100% and 1 pixel, respectively, and the corresponding MIG was 35. Thus, we set the MIG range to be 0–35, ignoring the exceeding part. Similarly, in the limiting case where the densities were set to be 20% and 85%, the corresponding ZSR values were 0.0817 and 0.011, respectively, where the size of speckle was 2 pixels. Thus, we set the ZSR range to be 0.01–0.08. We concluded that the quality of the speckle pattern was higher when CMZ was closer to zero.

## 4. Experimental Verification

### 4.1. CMZ Assessment Criterion

**Figure 4:**
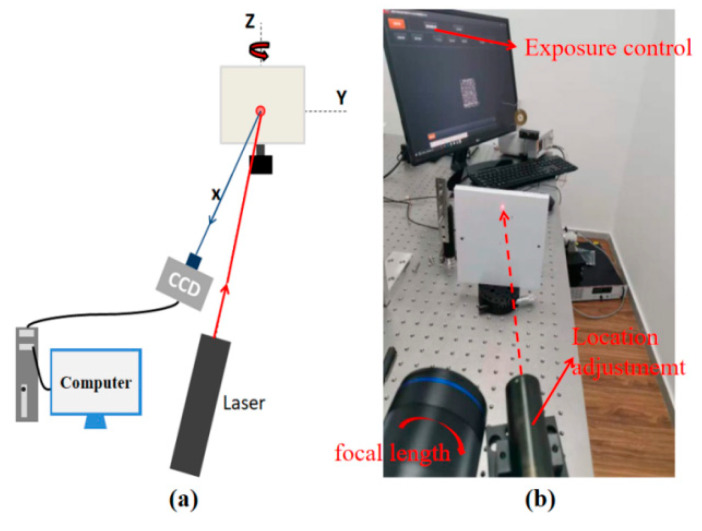
Experimental setup: (**a**) schematic of the experimental setup and (**b**) physical diagram of experimental setup.

To verify the effectiveness of the proposed assessment criterion based on the CMZ, experimental speckle patterns at different defocusing degrees, exposure times, and surface roughness were evaluated, and results were compared with the MIG and the MIOSD. As shown in Figure 4a, the object was irradiated by a laser beam, and then the surface of the object formed bright spots and dark spots due to coherent subwaves interference. The defocused speckle pattern was captured by a charge-coupled device (CCD) camera and analyzed by the computer. Figure 4b shows the experimental setup. The laser power of the He-Ne laser was 20 mW, and the wavelength was 632.8 nm. Defocusing degree related to speckle particle size was controlled by focal length. Exposure time related to gray contrast was adjusted through camera software. In order to ensure different roughness at different measurement locations, uniform white paint was sprayed on the surface of the specimen, and then sandpaper was used to polish different parts in different degrees.

The quality of the speckle pattern can be affected by factors: the defocusing degree, the camera exposure time, and the measurement location. The defocusing degree and the camera exposure time are related to speckle particle size and gray contrast, respectively, and the different measurement locations are due to the roughness difference.

**Figure 5:**
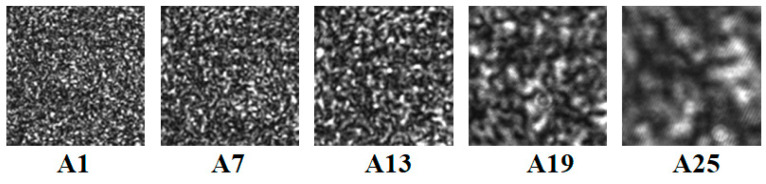
Speckle patterns collected at different defocusing degrees.

During speckle pattern acquisition, the defocusing degree was adjusted gradually, and the exposure time of the camera was then slightly adjusted around the value 8000 to retain the gray contrast. Twenty-eight speckle patterns (Group A: A1–A28, size: 256 × 256 pixels) with increasing speckle sizes were obtained, which are partially shown in Figure 5. In order to obtain deformed images with 0–1 pixel translation, sub-pixel shifted operation along the x-direction in the Fourier domain [34] was done. The step of translation was set to be 0.1 pixels. Decorrelation caused by big displacement or tilt should be avoided to ensure the validation of correlation calculation. Based on the DIC algorithm described in [46], the displacements of 81 points of each deformed image were calculated, and then the curves of the mean bias errors and the standard deviations of the displacements were obtained.

**Figure 6:**
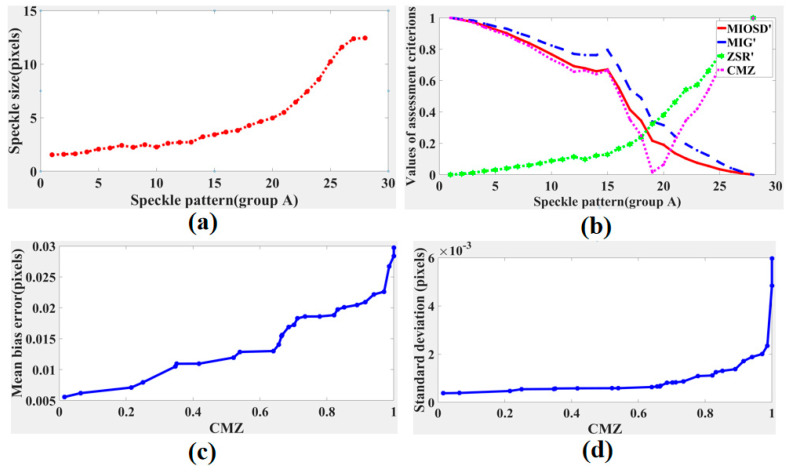
Results of speckle patterns in group A: (**a**) speckle size curve, (**b**) assessment criteria, (**c**) mean bias errors with different CMZ values, and (**d**) standard deviations with different CMZ values.

The average speckle size curve of the speckle pattern in group A is shown in Figure 6a. Results curves of MIG, MIOSD, ZSR, and CMZ assessment criteria are shown in Figure 6b. When sub-pixel translation is imposed to be 0.3 pixels, mean bias error curve of calculated displacement with different CMZ values is shown in Figure 6c, and standard deviation curve of calculated displacement with different CMZ values is shown in Figure 6d. The MIG and MIOSD values decreased as the speckle size increased. Conversely, the ZSR values increased as the speckle size increased. According to Figure 6b, the values of the proposed CMZ assessment criterion decreased first and then increased. Based on our research, the smaller the value of CMZ was, the higher the speckle pattern quality was. Thus, speckle patterns A15–A20, whose CMZ values were less than 0.3, were superior to the other patterns. As shown in Figure 6a, the speckle sizes of speckle patterns A15–A20 were between 3 and 5 pixels, which are consistent with the optimal speckle size during the DIC calculation. Results of Figure 6c,d verified that quality of the speckle pattern was higher when CMZ was closer to zero. Furthermore, standard deviation curve was relatively flat when CMZ was small.

**Figure 7:**
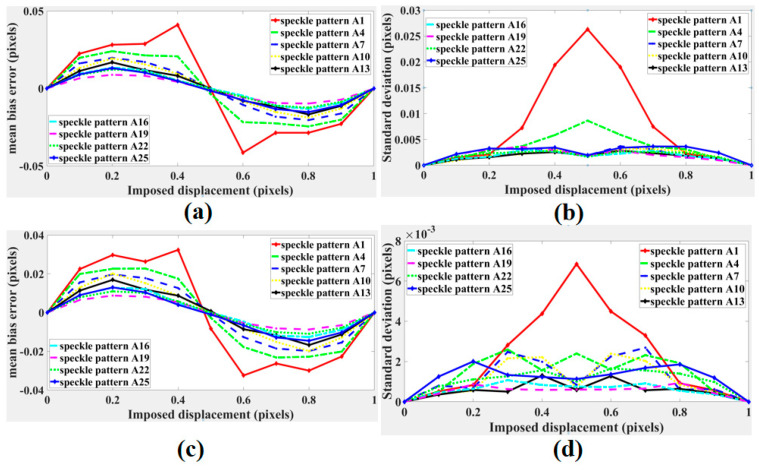
(**a**) Mean bias errors and (**b**) standard deviations of speckle patterns displacements in group A calculated with the subset of 31 × 31 pixels. (**c**) Mean bias errors and (**d**) standard deviations of speckle patterns displacements in group A calculated with the subset of 61 × 61 pixels.

The curves of the mean bias errors and standard deviations calculated with the subset of 31 × 31 pixels are shown in Figure 7a,c, respectively. The curves of the mean bias errors and standard deviations calculated with the subset of 61 × 61 pixels are shown in Figure 7b,d, respectively. The mean bias errors and standard deviations both decreased first and then increased, and the displacement calculation errors of pattern A19 were the lowest. The results were consistent with the results predicted by the proposed CMZ criterion. Furthermore, calculation with the subset of 61 × 61 pixels performed better. Therefore, choosing a larger subset can improve the calculation accuracy.

**Figure 8:**
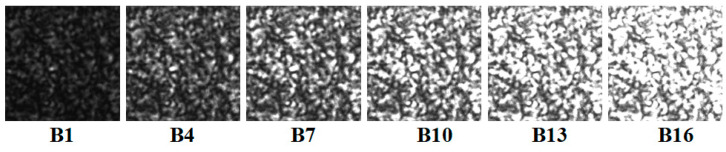
Speckle patterns collected at different camera exposure times.

To obtain speckle patterns with different gray contrasts, the defocusing degree was unchanged, and the exposure time of the camera was adjusted from 1000 to 16,000 gradually. Sixteen speckle patterns (Group B: B1–B16) were obtained, which are partly shown in Figure 8, and then the same translation operation and displacement error calculation were performed.

**Figure 9:**
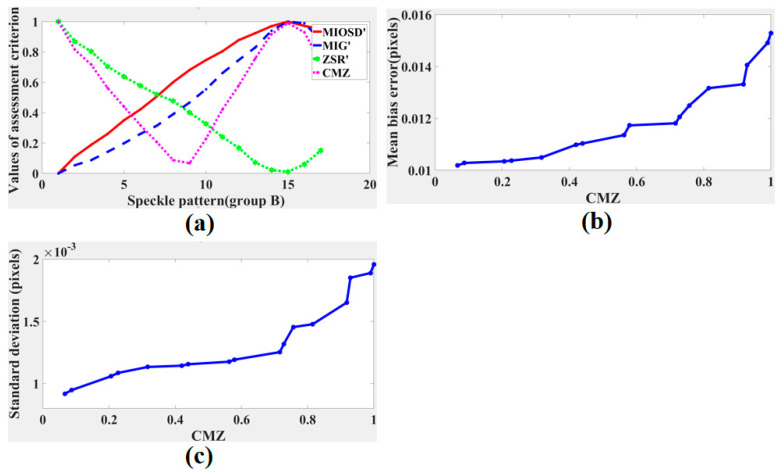
(**a**) Results of speckle patterns in group B: (**a**) assessment criteria, (**b**) mean bias errors with different CMZ values, and (**c**) standard deviations with different CMZ values.

The result curves of different assessment criteria are shown in Figure 9a. The MIG value increased and then decreased as the exposure time increased. According to the greater MIG principle, speckle patterns B13–B15 had stronger abilities to resist noise, which are supposed to have higher calculation accuracies. However, the proposed CMZ principle considers that the qualities of speckle patterns B6–B10 were superior. The trend of MIG and CMZ both indicate that the speckle pattern quality will decline when overexposure or underexposure because of decline of gray contrast. Mean bias error curve and standard deviation curve of calculated displacement with different CMZ values are drawn in Figure 9b,c, respectively. Mean bias error increased when CMZ value increased. Standard deviation and CMZ value also showed synchronous growth.

**Figure 10:**
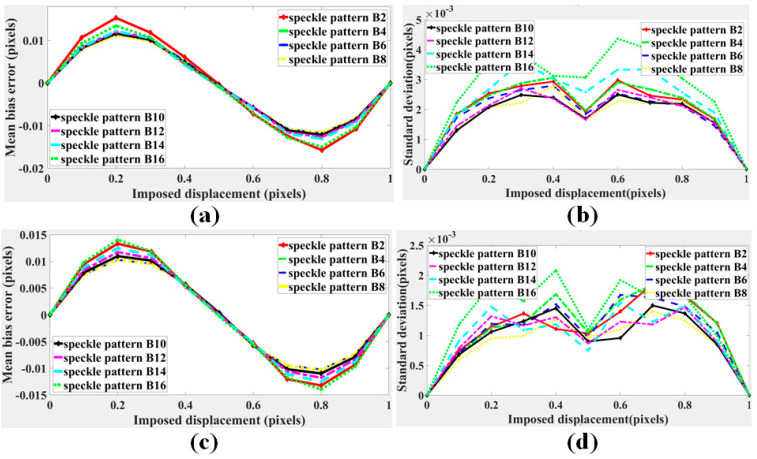
(**a**) Mean bias errors and (**b**) standard deviations of of speckle patterns displacements in group B calculated with the subset of 31 × 31 pixels. (**c**) Mean bias errors and (**d**) standard deviations of speckle patterns displacements in group B calculated with the subset of 61 × 61 pixels.

The curves of the mean bias errors and standard deviations calculated with the subset of 31 × 31 pixels are shown in Figure 10a,c, respectively. The curves of the mean bias errors and standard deviations calculated with the subset of 61 × 61 pixels are shown in Figure 10b,d, respectively. The mean bias errors and standard deviation both decreased first and then increased, which verified that the contrast of speckle patterns will be reduced due to overexposure or underexposure, causing decline of speckle pattern quality. According to displacement calculation errors, pattern B6–B10 perform better, which was closer to the result predicted by the proposed criterion CMZ. Similarly, calculation with the subset of 61 × 61 pixels performed better.

**Figure 11:**
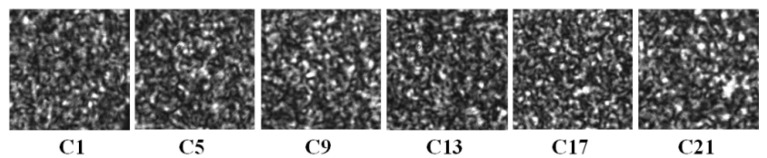
Speckle patterns collected at different measurement locations.

**Figure 12:**
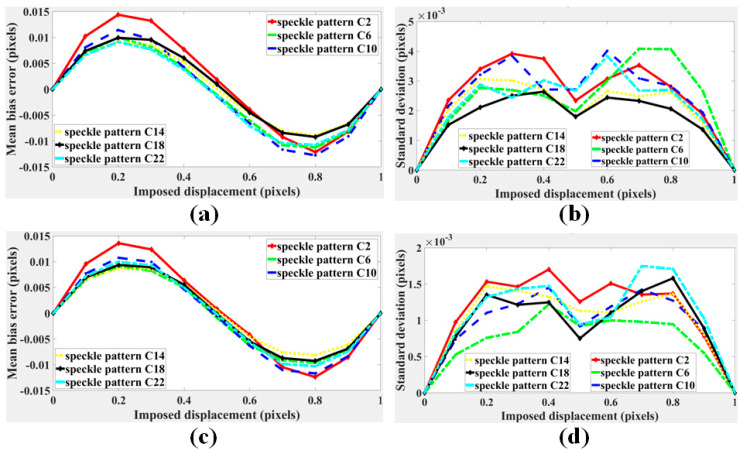
(**a**) Mean bias errors and (**b**) standard deviations of speckle patterns displacements in group C calculated with the subset of 31 × 31 pixels. (**c**) Mean bias errors and (**d**) standard deviations of speckle patterns displacements in group C calculated with the subset of 61 × 61 pixels.

To further evaluate the proposed CMZ assessment criterion, we changed the measurement locations to acquire speckle patterns C1–C25 with nearly the same gray contrasts and speckle sizes but different speckle particle location distributions due to the object surface roughness, as partially shown in Figure 11. The mean bias error and standard deviation curves calculated with the subset of 31 × 31 pixels are shown in Figure 12a,b, respectively, and those calculated with the subset of 61 × 61 pixels are shown in Figure 12c,d, respectively. The results of different speckle patterns were similar, and no trend was evident.

**Figure 13:**
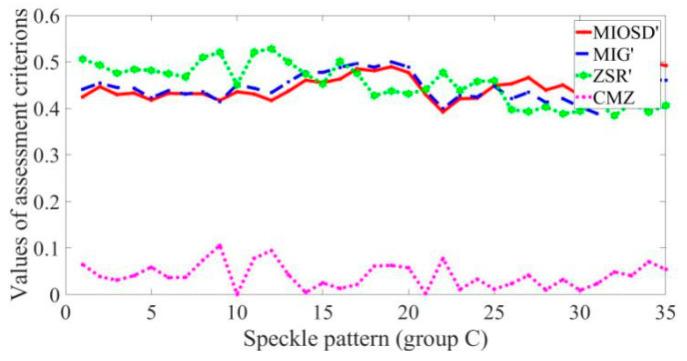
Comparison of different assessment criteria.

The results for different assessment criteria are shown in Figure 13. The MIG, MIOSD, ZSR, and CMZ values all remained stable, which is consistent with the displacement calculation results presented in Figure 12. Thus, the surface roughness of the object has little effect on its speckle pattern quality. High laser speckle pattern quality depends on suitable speckle size and gray contrast, which can be controlled by defocus degree and exposure time and so on. When the gray contrast is unchanged and the speckle size keeps within a suitable range, the displacement calculation accuracy of the laser speckle pattern remains stable with different speckle particle distributions.

### 4.2. Rotation Experiment

**Figure 14:**
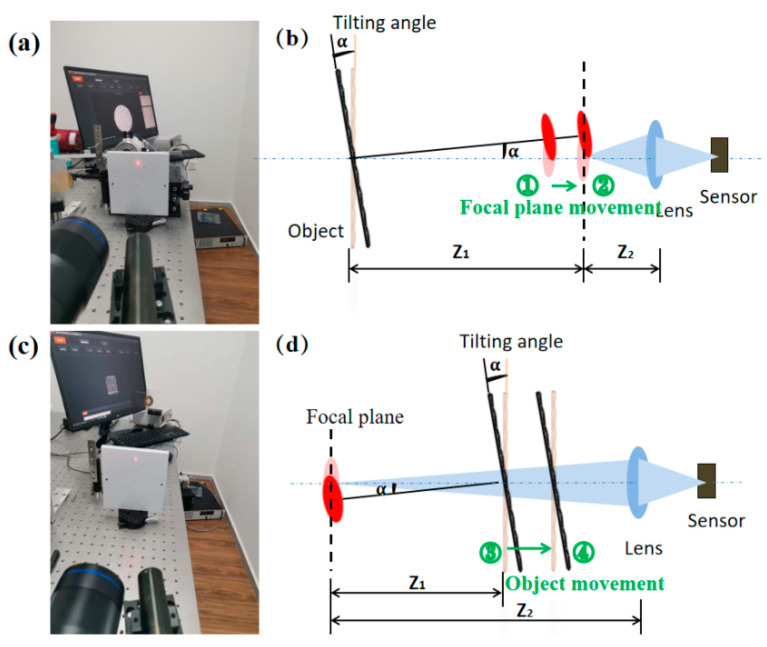
(**a**) Physical and (**b**) schematic diagrams of the first two experiments. (**c**) Physical and (**d**) schematic diagrams of the last two experiments.

**Figure 15:**
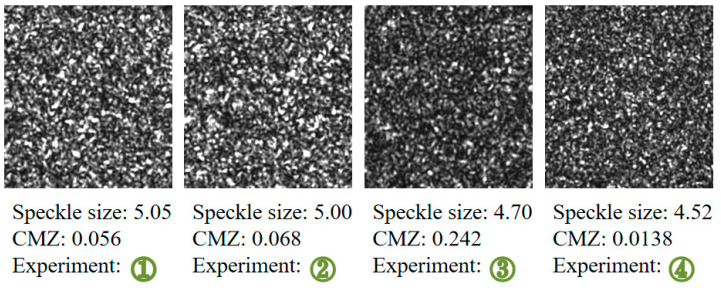
Four speckle patterns from the different experiments, their speckle sizes, and their CMZ values.

Considering the vibration information is obtained from tilt angle, rotation experiments were first designed to test angle calculation accuracy and further verify the proposed quality assessment. Besides, different relative distances between the object, the focal plane, and the sensor plane were adopted, which corresponded to different defocusing degree, so as to illustrate the relationship between defocusing degree and the angle resolution. The schematic diagram of the rotation experiment is shown in Figure 4a. A square measured plate was placed on a rotary platform, and the position accuracy was 2′. The plate was irradiated by a laser beam, and then the defocused speckle pattern was acquired by a CCD camera. The laser power of He-Ne laser was 20 mW, and the wavelength was 632.8 nm. We conducted four experiments. In two, the measured object was placed behind the focal plane, and in the other two, the measured object was placed in front of the focal plane, as shown in Figure 14a,c. In the two experiments with the object in front (shown in Figure 14b we changed the focal plane by adjusting the focal length slightly and keeping the other parameters fixed. In the two experiments with the object behind (shown in Figure 14d, only the location of the object changed. Based on the optimal speckle size determined by experience, the relative distances were first determined according to Equation (3) to ensure that the speckle sizes were all around 5 pixels. Next, the exposure time of the camera was adjusted to ensure a low CMZ value, because a low CMZ value corresponds to a high quality of the speckle pattern. The four speckle patterns given in Figure 15 are from the four different experiments. The speckle sizes and CMZ values of these speckle patterns were also calculated and are shown. The CMZ value of the third experiment was highest due to its low gray contrast. The CCD camera was a CP70 1HS M/C (Optronis, Germany). The physical size of the CCD sensor was 17.536 × 11.782 mm, and the size of the acquired speckle pattern was 1280 × 860 pixels.

**Figure 16:**
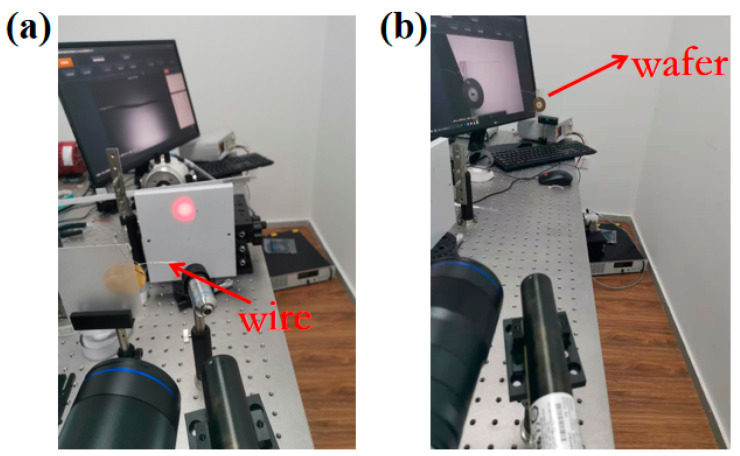
Physical diagram of focal plane determination in (**a**) the first two experiments and (**b**) the last two experiments.

As shown in Figure 16, we used an object with a known size, such as a wire or wafer, to find the focal plane where the acquired image was clearest. The camera magnification factor was calculated through the real width of the wire or wafer and its corresponding pixel width. The rotation angle resolution, which was defined as the calculated displacement at the focal plane when rotating 1′, could be obtained through the magnification factor and the distance between the object and the focal plane. In the four experiments, we adjusted the rotation angle to acquire different speckle patterns, and then displacement fields containing 100 calculated points at the focal plane were calculated by normal DIC algorithm. Mean displacement of each speckle pattern was eventually converted to the measured angle using Equation (4). The range of the rotation angle was 4’–20’, and the step of rotation was set to be 2’.

The measurement parameters and calculated angle resolutions are shown in Table 1. Comparing experiments 1 and 2, we found that when the distance between the sensor plane and object was fixed and the object was placed between the focal and sensor planes, the larger $Z_{1}$ corresponded to a larger angle resolution. According to experiments 3 and 4, when the magnification was fixed, $Z_{1}$ was larger, and the angle resolution was higher. Thus, the results are consistent with the analysis based on Equation (1).

**Figure 17:**
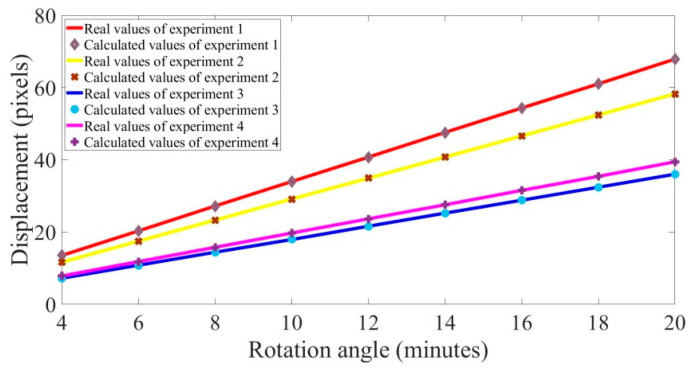
Comparison of displacement results at different rotation angles.

For all rotation angles, the real displacements at the focal plane are plotted as a line and the calculated displacements are plotted as scatter points in Figure 17, and results of the different experiments were all in good agreement. The angle resolution was considered to be the slope of the best fit line to the scatter points.

Table 2 gives the calculated slopes and errors. The calculated slopes were equal to the desired angle resolution. The mean errors of the displacements were less than 0.04 pixels, verifying the effectiveness of the laser speckle correlation method and further verifying the effectiveness of the CMZ speckle pattern quality criteria. Furthermore, the mean error and standard deviation of third experiment were the highest, which was consistent with the largest CMZ value of its speckle pattern. The mean errors of the rotation angle did not exceed 0.02 min, but the error included correlation calculation errors and calculated errors of the camera magnification.

### 4.3. Vibration Experiment

**Figure 18:**
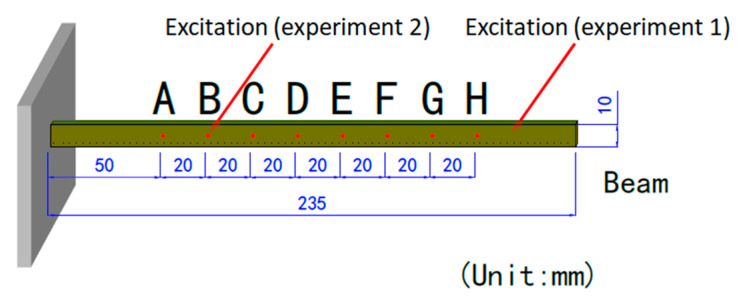
Dimension diagram of cantilever beam.

**Figure 19:**
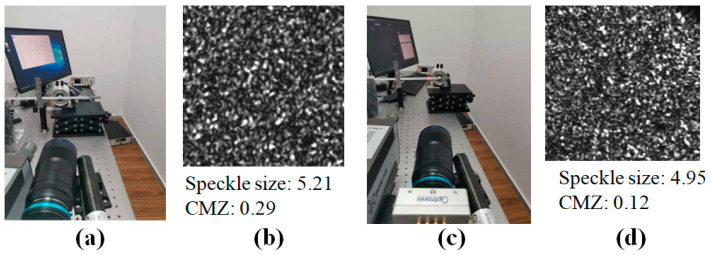
(**a**) Physical diagram of the first experiment. (**b**) One speckle pattern of the first experiment, its speckle size, and its CMZ value. (**c**) Physical diagram of the second experiment. (**d**) One speckle pattern of the second experiment, its speckle size, and its CMZ value.

Two real vibration experiments were conducted to further evaluate accuracy of the laser speckle correlation method. Using the same measurement system, a vibrating beam was used as the measured object. The vibration signal was passed through the signal generator, to the amplifier, and finally to the cantilever beam. To ensure that the speckle sizes were all around 5 pixels, the defocusing degree was determined using Equation (4), and then the exposure time was adjusted to ensure a low CMZ value to acquire a speckle pattern with a high quality. The size of the vibrating beam was 235 mm × 10 mm, as shown in Figure 18. Eight points (A–H) were measured in turn, where the distance from point A to the fixed end was 50 mm and the interval of the measurement points was 20 mm. The first experiment is illustrated in Figure 19a. At a distance of 30 mm from the free end, a simple harmonic excitation was applied to the cantilever beam with a frequency of 10 Hz, which was close to the first-order natural frequency of the beam. In the second experiment, which is shown in Figure 19c, the simple harmonic excitation was applied to point B with a frequency of 30 Hz. One speckle pattern coming from each experiment and the corresponding CMZ values are given in Figure 19b,d respectively. The laser power of the He-Ne laser was 20 mW, and the wavelength was 632.8 nm. However, the size of acquired speckle pattern was set to be 256 × 256 pixels to achieve a high sampling rate. The sampling rate was 2000 Hz, and the sampling time was 0.5 s. Meanwhile, laser doppler vibrometry (LDV) with a sampling rate of 40,000 Hz was used to collect the vibration signals. Finally, the calculated results of the speckle correlation method were compared with the signal processing results of the Doppler vibrator.

**Figure 20:**
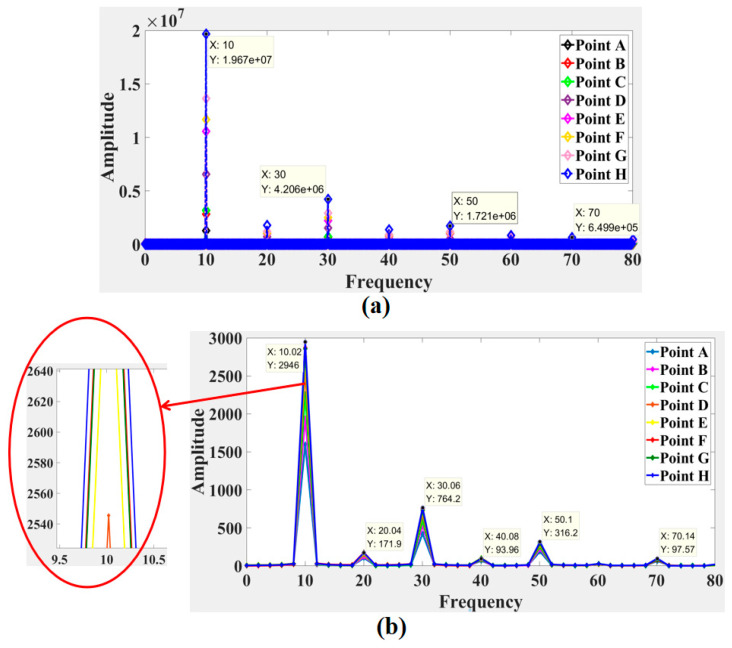
Comparison of the frequency spectrum obtained by (**a**) the LDV signals and (**b**) speckle patterns in the first experiment.

**Figure 21:**
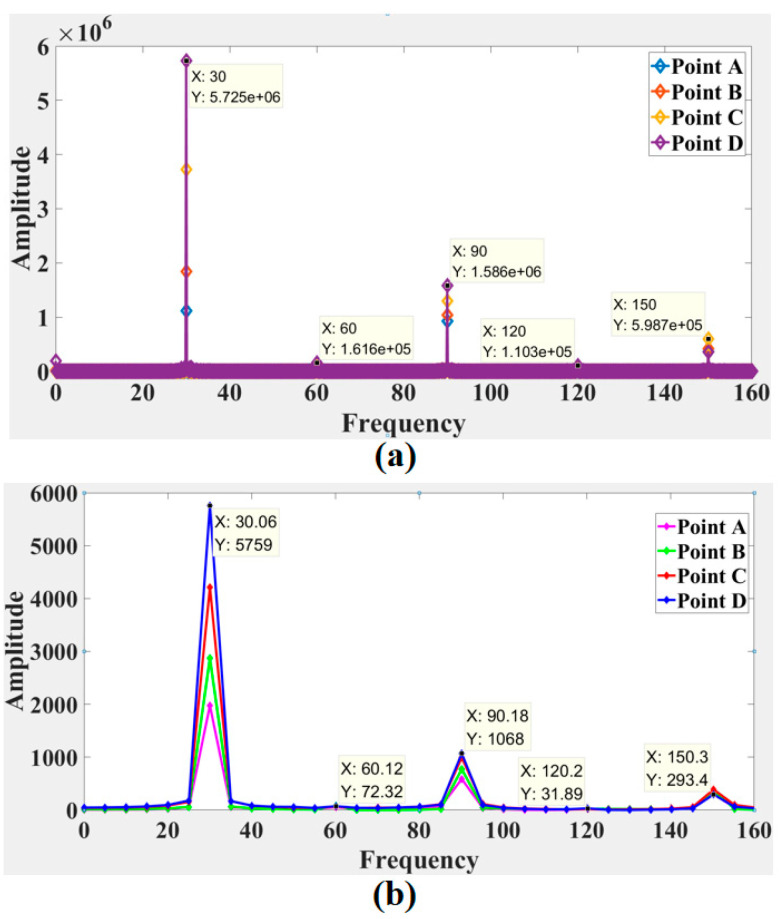
Comparison of the frequency spectrum obtained by (**a**) the LDV signals and (**b**) speckle patterns in the second experiment.

Figure 20a and Figure 21a show the frequency spectrum results from the two experiments obtained by the LDV, and Figure 20b and Figure 21b show the frequency spectrum results from the two experiments calculated by the laser speckle correlation method. The focus of this study was the response frequency, so the pixel displacement was not translated into a physical displacement. The calculated response frequencies of the two methods were in good agreement, and response frequencies showed no obvious difference at different tested location. In the frequency spectrum of the first experiment, a primary energy peak at 10 Hz. Several secondary energy peaks were present at 20 Hz, 30 Hz, 40 Hz, and 50 Hz. The response frequency error of the laser speckle correlation method increased from 0.02 Hz to 0.1 Hz. Similarly, the second experiment showed primary peak at 30 Hz. Secondary peaks happened at 90 and 150 Hz, respectively. The response frequency error of the proposed method increased from 0.06 Hz to 0.3 Hz.

In order to further verify the quality assessment criterion, excitation signal frequency was kept 30Hz, and defocusing degree was changed to acquire four groups of vibration laser patterns with different CMZ value. Response frequencies at point G were calculated and compared. Results were shown in Table 3.

From the results we can see, the frequency error increases with the CMZ value rising, verifying the effectiveness of the proposed quality assessment criterion.

## 5. Conclusions

To balance the random error and the interpolation bias, a global assessment criterion CMZ was proposed, which combined the MIG and the ZSR. A CMZ closer to zero corresponded to a better quality of the speckle pattern and a smaller displacement error. Considering that the laser speckle has a more uniform distribution of speckle particles, the simulated speckle patterns with different speckle particle sizes and densities were used to illustrate the determination of the CMZ criterion.

During the application of laser speckle correlation method, the main influencing factors include defocusing degree, exposure time and measured location. Experimental speckle patterns at different defocusing degrees, exposure times and measured locations were analyzed, and the results validated the proposed assessment criterion using the CMZ. Defocusing degree affects speckle particle size. The analysis results also further showed that the optimal speckle particle size was 3–5 pixels, and the contrast of the speckle patterns was reduced due to the overexposure, causing a decline of the speckle pattern quality. Thus, high quality speckle patterns can be guaranteed based on the CMZ criterion.

The resolution and accuracy of the laser speckle correlation method were found to be related to the distances between the measurement planes. In the experimental setup stage, a suitable defocusing degree can be determined based on the resolution requirement and the optimal speckle size, and then other experimental parameters can be determined according to the CMZ criterion. In rotation experiments, the comparison of the angle resolution verified the relationship between the resolution and distance, and the accuracy of the calculated displacement was consistent with the results predicted by the CMZ value. The frequency spectrum results of the vibration experiments were in good agreement with the LDV results, which simultaneously verified the effectiveness of the laser speckle correlation method and the reliability of the experimental setup based on the proposed CMZ rule. Thus, adopting this experimental setup method can ensure the resolution and accuracy of the laser speckle correlation method and facilitate its widespread application.

## Figures and Tables

**Table 1:** Comparison of measurement parameters and calculated angle resolutions.

Experiment	Object at Focus Plane (Pixels/mm)	M	Z1 (mm)	Z2 (mm)	Angle Resolution (Pixels/Minutes)
1	36	0.4932	324	150	3.393
2	32	0.4384	313	161	2.913
3	9	0.1233	730	1200	1.800
4	9	0.1233	754	1200	1.971

Notice: (1 pixels/minute = 1 pixels/$\frac{\pi }{180\times 60}\mathrm{rad}$).

**Table 2:** Comparison of error results from different experiments.

Experiment	Calculated Slope (Pixels/Minutes)	Displacement at Focus Plane (Pixels)		Rotation Angle (Minutes)	
		Mean Error	Standard Deviations	Mean Error	Standard Deviations
1	3.393	0.023	0.008	0.017	0.0064
2	2.913	0.030	0.012	0.017	0.0042
3	1.800	0.036	0.009	0.020	0.0053
4	1.971	0.021	0.011	0.006	0.0034

**Table 3:** Frequency results comparison of different pattern groups.

Patterns Group	CMZ Value	Frequency (Hz)		
		Primary Energy Peak	Secondary Energy Peak	Secondary Energy Peak
1	0.06	30.01	90.07	150.09
2	0.12	30.03	90.11	150.17
3	0.29	30.06	90.18	150.30
4	0.45	30.19	90.35	150.75

## Data Availability

Not applicable.

## References

[1] Huang M., He Z.-N., Lee F.-Z. (2004). A novel methodology for enhancing the contrast of correlation fringes obtained by ESPI. Measurement.

[2] Wang S., Lu M., Bilgeri L.M., Jakobi M., Bloise F.S., Koch A.W. (2018). Temporal electronic speckle pattern interferometry for real-time in-plane rotation analysis. Opt. Express.

[3] Barile C., Casavola C., Pappalettera G., Pappalettere C. (2014). Analysis of the effects of process parameters in residual stress measurements on Titanium plates by HDM/ESPI. Measurement.

[4] Pagliarulo V., Farroni F., Ferraro P., Lanzotti A., Martorelli M., Memmolo P., Speranza D., Timpone F. (2018). Combining ESPI with laser scanning for 3D characterization of racing tyres sections. Opt. Lasers Eng..

[5] Falldorf C., Agour M., Bergmann R.B. (2015). Digital holography and quantitative phase contrast imaging using computational shear interferometry. Opt. Eng..

[6] Wang X., Gao Z., Yang S., Gao C., Sun X., Wen X., Feng Z., Wang S., Fan Y., Wang X. (2018). Application of digital shearing speckle pattern interferometry for thermal stress. Measurement.

[7] Frankovský P., Brodnianská Z., Bocko J., Trebuňová M., Kostka J., Kicko M., Čarák P. (2020). Application of holographic interferometry in the analysis of stress states in a crack root area. Appl. Opt..

[8] Abdelsalam D. (2013). A comparison of digital holographic microscopy and on-axis phase-shifting interferometry for surface profiling. Measurement.

[9] Hartlieb S., Tscherpel M., Guerra F., Haist T., Osten W., Ringkowski M., Sawodny O. (2021). Highly accurate imaging based position measurement using holographic point replication. Measurement.

[10] Huang T., Li X., Fu X., Zhang C., Duan F., Jiang J. (2019). Arbitrary phase shifting method for fiber-optic fringe projection profilometry based on temporal sinusoidal phase modulation. Opt. Lasers Eng..

[11] Hyun J.-S., Zhang S. (2016). Enhanced two-frequency phase-shifting method. Appl. Opt..

[12] Gu G., Pan Y., Qiu C., Zhu C. (2021). Real-time dual-channel speckle interferometry based on an improved dual-observation configuration with spatial phase-shifting. Measurement.

[13] Liu C.-H., Schill A., Raghunathan R., Wu C., Singh M., Han Z., Nair A., Larin K.V. (2017). Ultra-fast line-field low coherence holographic elastography using spatial phase shifting. Biomed. Opt. Express.

[14] Kim J.A., Kim J.W., Kang C.S., Jin J., Eom T.B. (2018). Interferometric profile scanning system for measuring large planar mirror surface based on single-interferogram analysis using Fourier transform method. Measurement.

[15] Prabhakar D., Kumar M.S., Krishna A.G. (2020). A Novel Hybrid Transform approach with integration of Fast Fourier, Discrete Wavelet and Discrete Shearlet Transforms for prediction of surface roughness on machined surfaces. Measurement.

[16] Fu Y., Guo M., Phua P.B. (2010). Spatially encoded multibeam laser Doppler vibrometry using a single photodetector. Opt. Lett..

[17] Fu Y., Guo M., Phua P.B. (2011). Multipoint laser Doppler vibrometry with single detector: Principles, implementations, and signal analyses. Appl. Opt..

[18] Halkon B.J., Rothberg S.J. (2020). Establishing correction solutions for Scanning Laser Doppler Vibrometer measurements affected by sensor head vibration. Mech. Syst. Signal Process..

[19] Sciuti V.F., Canto R.B., Veggers J., Hild F. (2021). On the benefits of correcting brightness and contrast in global digital image correlation: Monitoring cracks during curing and drying of a refractory castable. Opt. Lasers Eng..

[20] Wang W., Mottershead J.E., Siebert T., Pipino A. (2012). Frequency response functions of shape features from full-field vibration measurements using digital image correlation. Mech. Syst. Signal Process..

[21] Rosakis A.J., Rubino V., Lapusta N. (2020). Recent Milestones in Unraveling the Full-Field Structure of Dynamic Shear Cracks and Fault Ruptures in Real-Time: From Photoelasticity to Ultrahigh-Speed Digital Image Correlation. J. Appl. Mech..

[22] Hu X., Xie Z., Liu F. (2021). Assessment of speckle pattern quality in digital image correlation from the perspective of mean bias error. Measurement.

[23] Chen Z., Quan C., Zhu F., He X. (2015). A method to transfer speckle patterns for digital image correlation. Meas. Sci. Technol..

[24] Song J., Yang J., Liu F., Lu K. (2018). High temperature strain measurement method by combining digital image correlation of laser speckle and improved RANSAC smoothing algorithm. Opt. Lasers Eng..

[25] Zheng Q., Mashiwa N., Furushima T. (2020). Evaluation of large plastic deformation for metals by a non-contacting technique using digital image correlation with laser speckles. Mater. Des..

[26] Gregory D. (1976). Basic physical principles of defocused speckle photography: A tilt topology inspection technique. Opt. Laser Technol..

[27] Hrabovsky M.H., Šmíd P. (2004). Full theory of speckle displacement and decorrelation in the image field by wave and 27. Pgeometrical descriptions and its application in mechanics. J. Mod. Opt..

[28] Jo K., Mohit G., Shree K.N. Spedo: 6 dof ego-motion sensor using speckle defocus imaging. Proceedings of the IEEE International Conference on Computer Vision.

[29] Zalevsky Z., Beiderman Y., Margalit I., Gingold S., Teicher M., Mico V., Garcia J. (2009). Simultaneous remote extraction of multiple speech sources and heart beats from secondary speckles pattern. Opt. Express.

[30] Li L., Gubarev F.A., Klenovskii M.S., Bloshkina A.I. Vibration measurement by means of digital speckle correlation. Proceedings of the 2016 International Siberian Conference on Control and Communications (SIBCON).

[31] Beiderman Y., Horovitz I., Burshtein N., Teicher M., García J., Micó V., Zalevsky Z. (2010). Remote estimation of blood pulse pressure via temporal tracking of reflected secondary speckles pattern. J. Biomed. Opt..

[32] Wu N., Haruyama S. (2020). Real-time audio detection and regeneration of moving sound source based on optical flow algorithm of laser speckle images. Opt. Express.

[33] Sun Y.F., Pang J.H.L. (2007). Study of optimal subset size in digital image correlation of speckle pattern images. Opt. Lasers Eng..

[34] Pan B., Xie H., Wang Z., Qian K., Wang Z. (2008). Study on subset size selection in digital image correlation for speckle patterns. Opt. Express.

[35] Lecompte D., Smits A., Bossuyt S., Sol H., Vantomme J., Van Hemelrijck D., Habraken A. (2006). Quality assessment of speckle patterns for digital image correlation. Opt. Lasers Eng..

[36] Crammond G., Boyd S., Dulieu-Barton J. (2013). Speckle pattern quality assessment for digital image correlation. Opt. Lasers Eng..

[37] Pan B., Lu Z.X., Xie H.M. (2010). Mean intensity gradient: An effective global parameter for quality assessment of the speckle patterns used in digital image correlation. Opt. Lasers Eng..

[38] Yu H., Guo R., Xia H., Yan F., Zhang Y., He T. (2014). Application of the mean intensity of the second derivative in evaluating the speckle patterns in digital image correlation. Opt. Lasers Eng..

[39] Park J., Yoon S., Kwon T.-H., Park K. (2017). Assessment of speckle-pattern quality in digital image correlation based on gray intensity and speckle morphology. Opt. Lasers Eng..

[40] Bossuyt S. (2013). Optimized patterns for digital image correlation. Imaging Methods for Novel Materials and Challenging Applications.

[41] Stoilov G., Kavardzhikov V., Pashkouleva D. (2012). A Comparative Study of Random Patterns for Digital Image Correlation. J. Theor. Appl. Mech..

[42] Bomarito G., Hochhalter J., Ruggles T., Cannon A. (2017). Increasing accuracy and precision of digital image correlation through pattern optimization. Opt. Lasers Eng..

[43] Song J., Yang J., Liu F., Lu K. (2020). Quality assessment of laser speckle patterns for digital image correlation by a Multi-Factor Fusion Index. Opt. Lasers Eng..

[44] Su Y., Zhang Q., Gao Z., Xu X., Wu X. (2015). Fourier-based interpolation bias prediction in digital image correlation. Opt. Express.

[45] Charrett T., Tatam R. (2019). Performance and Analysis of Feature Tracking Approaches in Laser Speckle Instrumentation. Sensors.

[46] Pan B. (2013). An Evaluation of Convergence Criteria for Digital Image Correlation Using Inverse Compositional Gauss-Newton Algorithm. Strain.

